# Beyond implementation: what drives long-term sustainment of life-sustaining treatment documentation in VA home based primary care

**DOI:** 10.1186/s12913-026-15043-7

**Published:** 2026-07-09

**Authors:** Jennifer Kononowech, Kate H. Magid, Leah M. Haverhals, Jazmin Beltran, Cari R. Levy

**Affiliations:** 1 Center for Clinical Management Research, VA Ann Arbor Healthcare System, Ann Arbor, MI USA; 2 Denver-Seattle VA Center of Innovation for Value Driven and Veteran Centric Care, Rocky Mountain Regional VA Medical Center at VA Eastern Colorado Health Care System, Aurora, CO USA; 3 Division of Geriatric Medicine, University of Colorado Anschutz Medical Campus, Aurora, CO USA

**Keywords:** Sustainability, Evidence-based practice, Long-term care

## Abstract

**Background:**

Sustaining evidence-based practices in health care systems remains a persistent challenge. While many implementation efforts successfully achieve adoption and short-term outcomes, these gains may diminish once external support is withdrawn. Understanding organizational and contextual factors influencing sustainability is necessary for long-term success. We designed the Preferences Elicited and Respected for Seriously Ill Veterans (PERSIVED) United States Department of Veterans Affairs (VA) quality improvement project to support documentation of life-sustaining treatment (LST) preferences aimed to identify and explain determinants impacting sustainment following the end of active implementation strategies.

**Methods:**

Eleven Home-Based Primary Care (HBPC) programs participated in the stepped-wedge design that included pre-implementation, implementation, and sustainability phases. During the implementation phase, programs received monthly coaching and audit with feedback showing rates of LST documentation. During sustainability, coaching calls ceased with audit with feedback continuing. To explore contextual determinants of sustainability, semi-structured group interviews were conducted with site champions and leadership at months 4 and 12 of sustainability. Qualitative data were analyzed using a matrixed multiple case study approach guided by the Clinical Sustainability Assessment Tool.

**Results:**

All participating sites sustained or exceeded LST documentation rates reached during the implementation phase throughout the sustainability phase. Enabling factors included embedding LST documentation into clinical visit processes, viewing documentation as standard of care, clear team expectations, use of feedback reports to motivate change, and inclusion of LST documentation in provider performance evaluations. Differentiating factors included leadership engagement, mechanisms for monitoring performance data, and team awareness and beliefs regarding the importance of LST documentation. Hindering factors included staff shortages, competing demands, and limited time to engage in conversations regarding preferences and time to document preferences. Program expansion had a mixed impact depending on staffing capacity and workflow integration. Continuing education and use of PERSIVED resources had limited impact on sustainability.

**Conclusions:**

Embedding LST documentation into routine workflows, maintaining visibility of performance data, and reinforcing expectations by leadership and organizational processes led to sustainment of LST documentation in VA HBPC programs. These findings inform strategies for future studies emphasizing systems change and encourage the examination of sustainability over a longer period to assess which determinants are most influential for maintaining evidence-based practices after implementation ends.

**Supplementary Information:**

The online version contains supplementary material available at https://doi.org/10.1186/s12913-026-15043-7.

## Background

Sustaining evidence-based practices in health care systems is challenging [1]. Implementation efforts often succeed in achieving short-term outcomes and adoption of innovations, but without intentional strategies to support long-term sustainment, these gains may diminish once external resources are withdrawn [2]. As such, sustainability has emerged as a central concern for implementation science, emphasizing the need to understand the organizational and contextual factors that influence whether an evidence-based practice is sustained over time [3].

In 2017, the National Center for Ethics in Health Care (NCEHC) within the Department of Veterans Affairs (VA) implemented the Life-Sustaining Treatment Decisions Initiative (LSTDI) [4]. The LSTDI standardized processes for conducting goals of care conversations (GoCCs) and documenting Veterans’ life-sustaining treatment (LST) preferences in their electronic medical records through a standardized note template, the LST template. We developed the Preferences Elicited and Respected for Seriously Ill Veterans through Enhanced Decision Making (PERSIVED) quality improvement program to support uptake of the evidence-based practice of conducting GoCCs and documenting LST preferences in the VA LST template for Veterans receiving VA Home-Based Primary Care (HBPC) [5]. Veterans enrolled in HBPC are often frail with multiple comorbidities and receive care in their home from a multidisciplinary team of VA clinicians [6].

**Fig. 1:**
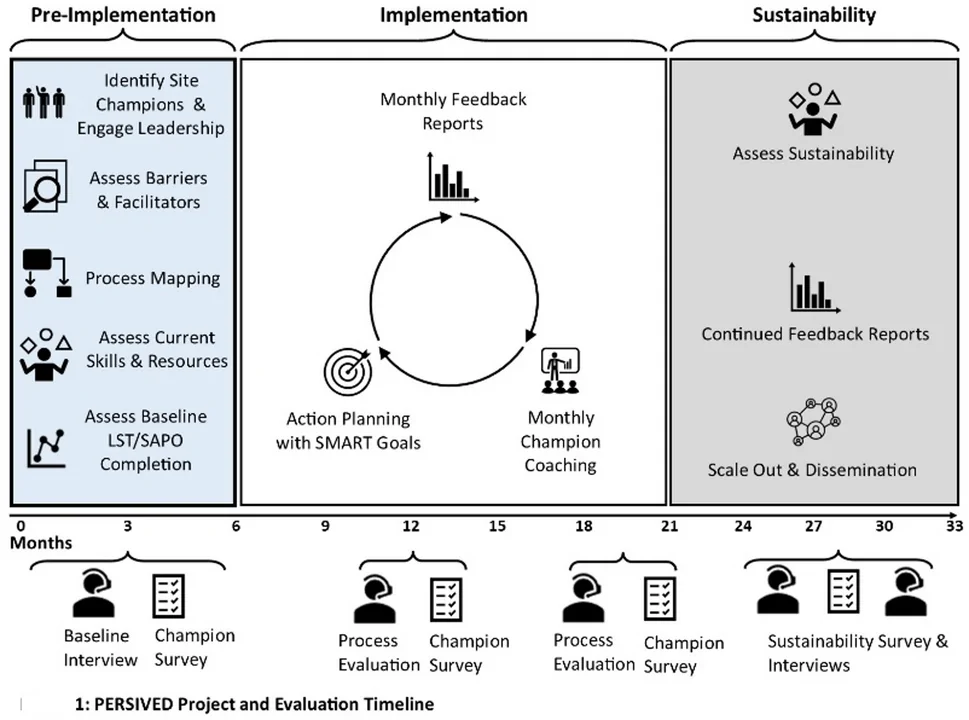
PERSIVED intervention

PERSIVED employed a 15-month implementation facilitation and audit with feedback intervention to support HBPC clinicians in increasing documentation of LST preferences in the electronic medical record (see Fig. 1: PERSIVED Intervention). During the implementation phase, participating programs achieved high rates of LST template documentation, demonstrating successful practice change [7]. Following implementation, PERSIVED programs entered a 1-year sustainability period during which implementation facilitation ceased but audit with feedback continued. Once these external resources were withdrawn, we aimed to identify what facilitated programs sustaining practice changes and improvements.

Prior work on sustainability has shown that long-term maintenance of evidence-based practices depends on organizational integration, leadership engagement, and involvement of local change agents [8]. Multiple studies within VA have demonstrated that practices are more likely to be sustained when they are embedded into routine clinical workflows, aligned with national and facility-level priorities, and supported by leadership [9–11]. In contrast, sustainability challenges have been linked to competing organizational priorities, staff turnover, lack of protected time to support sustainment, and lack of ongoing support [12].

We sought to identify determinants of sustainability in LST documentation one year after implementation. Specifically, we examined which factors acted as enabling, hindering, or had limited impact on sustainability, and how these factors varied across programs with steady or high rates of LST documentation during sustainability.

## Methods

### Overview

We invited HBPC programs to participate in PERSIVED if less than 50% of their Veteran program census had a documented LST template. The PERSIVED team reviewed a list of eligible programs with national VA HBPC leadership to ensure programs were appropriate for participation. A total of eleven HBPC programs enrolled in PERSIVED using a stepped-wedge design. PERSIVED began with a 6-month pre-implementation phase in which HBPC team members and leadership were interviewed to identify barriers and facilitators to LST documentation [5]. Interview data from the pre-implementation phase was used to inform our implementation facilitation strategies. Champions were identified from the HBPC programs and included clinicians, nurses, social workers, and program leadership (Table 1: Champion and Site Characteristics). We engaged at least two clinical champions per participating program. During the 15-month implementation phase, champions participated in monthly coaching calls with PERSIVED coaches with expertise on GoCCs and LST documentation, and received audit with feedback reports. During the 12-month sustainability phase, monthly coaching calls ended and feedback reports continued to be sent.

**Table 1:** Champion and Site Characteristics

Champion Characteristic	*N* (%)
	*N* = 32
***Race***	
Asian (including Hawaiian or Pacific Islander)	1 (3)
Black or African American	4 (13)
White	18 (56)
Multiracial	2 (6)
Missing/ Did not disclose	7 (22)
***Ethnicity***	
Hispanic	2 (6)
Non-Hispanic	23 (72)
Missing/ Did not disclose	7 (22)
***Role***	
Registered Nurse	7 (22)
Social Worker	6 (19)
Psychologist	2 (6)
Local Program Leadership	3 (9)
Local Program Leadership and Provider (MD, NP, PA)	3 (9)
Provider	11 (34)
***Time in Role at VA (years)***	
<1	3 (9)
1–3	9 (28)
4–9	11 (34)
10+	6 (19)
Missing/ Did not disclose	3 (9)
**Site Characteristic**	*N* = 11
***Total Number of Champions per Site***	
2	6
3	2
4	1
5	2

During the implementation phase, programs aimed to complete LST template documentation for at least 80% of the Veterans in their HBPC program.

We then assessed sustainability over a 12-month follow-up period, defining sustainability as stable (completion rates within 5% of the highest implementation rate) or high (>/ 6% above the highest implementation rate).

The VA Research Office at the Eastern Colorado Health Care System reviewed PERSIVED and determined the program to be a non-research, quality improvement project that did not require institutional board review or ethics approval.

## Data collection

### Quantitative data

We extracted site-level LST completion rates using the VA Corporate Data Warehouse (CDW) which is an information repository and includes all Veteran health data. Specific data elements, called LST health factors, were retrieved from the CDW to identify Veterans with a documented LST template. These data were merged with HBPC census data using the HBPC Master File.

### Qualitative data

To explore contextual factors influencing sustainability, we conducted group interviews with site champions and leadership from 10 HBPC programs during months 4 (*n* = 10) and 12 (*n* = 10) of sustainability. One HBPC program ceased participation during the implementation phase and did not complete sustainability interviews. Interviews were conducted using a semi-structured interview guide developed for this study (Additional File 1: Interview Guides). Two PERSIVED team members attended each group interview, with one serving as the main interviewer and the other as the secondary interviewer and notetaker. All interviews were conducted via video and/or audio using Microsoft Teams, with participants providing verbal consent to be recorded.

### Data analysis

Researchers developed the Clinical Sustainability Framework, operationalized in the Clinical Sustainability Assessment Tool (CSAT), to identify organizational and contextual domains influencing sustainability [13]. The CSAT includes seven domains that influence whether a clinical practice can be maintained: engaged staff and leadership; engaged partners; monitoring and evaluation; outcomes and effectiveness; organizational readiness; implementation and training; and workflow integration.

We based our analytic approach on work by Kim et al. which utilized a matrixed multiple case (MMC) study approach to understand processes and influences impacting outcomes across multiple programs [14]. The CSAT guided our analysis. We selected six of the seven CSAT domains that were most relevant to the sustainability context for PERSIVED: engaged staff and leadership; monitoring and evaluation; implementation and training; workflow integration; outcomes and effectiveness; and organizational readiness (see Additional File 2: PERSIVED Analytic Plan).

Using Atlas.ti software we generated facilitator and barrier code summaries for each site [15]. Analysts created co-occurrence queries on Atlas with the CSAT domain code groups and the facilitator and barrier codes. We also created code summaries for distinct codes (good quote, feedback reports, incentives, sustainability plan, priority) that were not commonly coded as facilitator or barrier.

To systematically assess site-level determinants, we created an Excel matrix with CSAT domains as rows and PERSIVED sites as columns. The matrix allowed structured comparison of the presence and valence of sustainability factors across sites.

Two analysts independently reviewed code summaries for each site and indicated the presence and influence of each factor, using a standardized rubric. Presence was rated from minimally present to present, and valence was rated from hindering to enabling. The two analysts met to reconcile discrepancies and achieve consensus coding on all interviews. This process enhanced reliability and ensured analytic rigor consistent with qualitative research standards (STaRI) [16].

Once site matrices were finalized, we explored determinants of sustainability across sites using the following categories:

1. Enabling factors: factors consistently facilitating sustainability across sites.
2. Differentiating factors: which factors or valence were linked to higher vs. lower LST documentation rates across sites.
3. Hindering factors: factors consistently identified as barriers to sustainability across sites.
4. Limited/no impact and mixed factors: determinants with limited to no impact on sustainability or that were mixed across sites.
  We compared factors across CSAT domains to identify domain level trends, assess whether certain determinants were emphasized more at some sites than others, and evaluate which combination of factors were most strongly linked to sustainability outcomes.

## Results

### Enabling

We identified several determinants that enabled LST documentation during the sustainability phase of PERSIVED. These *universal enablers* consistently facilitated LST template completion when present across sites. Embedding LST documentation into visit processes emerged as a *universal enabler*. Sites described that completion became routine once LST documentation was incorporated into admissions or visit processes. One provider shared:

It’s a standard part of our admission. So, when we put in our note, we automatically do the LST. Now it’s just become automatic, essentially. And then, of course on the annuals we do one if they don’t have them.

Viewing LST documentation as a standard of care proved to be another universal enabler. Sites that shared it was part of their normal processes and viewed it as automatic had high rates of LST documentation. One provider noted:

*It’s been integrated into our routine practice*,* and I don’t see that being any additional barrier or stopping [completing documentation]. I think it’s just part of our routine.*

A consistent expectation that all team members complete LST documentation further reinforced sustainability across sites. One HBPC team member shared:

I think we can only get better, like even, you know, now that we are again making it public that that’s an expectation that we’re having these conversations with people that we’re documenting these documents in the chart.

Reviewing and sharing the feedback reports created and delivered by the PERSIVED team also were identified as universally enabling factors. Sites explained that they shared feedback reports during provider and team meetings, and used them to set new goals for completion rates.

[We] present it every month in the staff meeting. So, we’re still doing that. So, it’s helpful so everybody knows where we are and you know, takes pride in it and you know continue to keep up the good work.

HBPC programs that included LST template documentation as part of their VA Ongoing Professional Practice Evaluation (OPPE) requirements found this to be universally enabling. One site shared:

*A new change was the addition of the LST on OPPE. So*,* I think that has been one of the drivers for the high rates of completion.*

HBPC programs can add items to their OPPE requirements, and those that chose to include LST documentation believed it contributed to higher rates of LST documentation.

Additionally, there were several factors identified as *mostly enabling* during the sustainability phase of PERSIVED. These factors generally facilitated ongoing completion of LST documentation when present, but didn’t have a negative impact when not present. Such determinants included the priority of LST documentation. Sites that viewed LST documentation as a priority indicated that it became a high priority for their entire team because they believed LST documentation was needed for their older, comorbid Veteran population, and wanted to ensure that Veterans’ wishes were documented.

*It’s* [LST documentation] *on the top right there because of the patient population we take care of*,* I think it’s actually a pretty important aspect of the care. So yeah*,* I would consider it one of the most important.*

Sites that indicated that LST documentation was less of a priority, stated that they had more urgent demands, including inability to hire needed staff.

Including information about LST templates and documentation during new employee training was also *mostly enabling* of template completion. Sites that included information about LST template completion during new employee training focused on providers and nurses, as providers complete the documentation and nursing staff are often involved in GoCCs.

For new hires, and mainly all new hires who are NPs or providers and nurses, because nurses and all the other team members are instrumental as well in getting those completed because they are an extra set of eyes and ears and they give us feedback on if there are any modifications that are needed, and you know, they can let us know if there’s maybe one or two that we may have missed that needs to be completed.

Discussing LST documentation during HBPC interdisciplinary team (IDT) meetings was also a *mostly enabling* factor. A number of sites kept LST documentation as a standing item on their IDT meeting agenda and tracked whether LST templates had been completed when discussing each Veteran. A site champion shared:

*I think the success in that was we included this in our IDT. So*,* every time a patient was reviewed quarterly or annually*,* it was part of the discussion. And so*,* there was this… repeated reminder that it needed to be done.*

One site with high rates of LST documentation at the end of sustainability shared that it had been a standing IDT agenda item during implementation, but was removed during the sustainability phase, which they viewed as a deficit.

### Differentiating

*Differentiating factors* were those that when present were enabling, but when absent hindered sustainment. Participants identified leadership support as a *differentiating factor.* Sites shared that some leadership actively engaged in supporting LST documentation by discussing it during IDT and provider meetings and stressing the importance of documentation.

It’s talked about so often that we can’t forget it because [name omitted], she keeps it keeps it going. Yeah, she keeps it going.

Other sites shared that leadership was not involved in LST template completion nor in talking about it during IDT and provider meetings, which lowered the priority of template completion and hindered sustainment.

When planning for the sustainability phase, the PERSIVED team talked with champions about creating a team monitoring plan to continue to review their LST documentation data. Some programs made spreadsheets to track LST documentation, reviewing these during team meetings.

*We still got our system going. We developed uh*,* a spreadsheet to keep up with our LST orders and each provider has access to it to add the names of the patients that you know*,* the new incoming patients.*

Other programs did not have processes or staff in place to monitor these data, and these sites believed this was a hindering factor for sustainability efforts.

*I think the problem that we could potentially run into is if the process stopped working as well. We don’t really have a system at this point to highlight that. And create a corrective action.*

Team awareness and beliefs also emerged as *differentiating factors*. Successful sites viewed LST documentation as an accepted practice and had buy-in from their teams. These sites believed they would continue to have high rates of documentation during the sustainability phase.

I believe we’re gonna continue to be [have] very high numbers. I mean, I would see us continuing to be in the 90th percentile somewhere, you know with completions because that continues to be an important measure that we need to address and complete.

Sites with varying awareness and doubts about their abilities to sustain documentation rates during the sustainability phase were hindered by these beliefs.

### Hindering

When not present, adequate staffing hindered sustainment across sites. Champions noted that staffing shortages led to increased workloads on remaining staff, which reduced their time to prioritize tasks such as completing LST templates due to more pressing needs and crises. As one provider champion shared:

*When we are short-staffed and we are trying to put out fires every day*,* sometimes things like that [LST templates] may get pushed down a little bit.*

When asked about changes to their team during sustainability, multiple sites described growth in patient census and facing challenges when hiring additional staff to meet the needs of the expanding patient population. A subset of these sites who experienced program expansion detailed challenges covering the increased patient caseload, particularly when paired with challenges filling vacant staff positions or hiring additional staff. Consequently, experiencing program expansion was either a hindering or neutral factor for sustainment when present. A provider at a site that expanded to a new geographic area described how the intersection of program growth and staff turnover increased staffing workloads, which made LST template completion challenging to fit in. They said:

We’ve been trying to back fill [Provider’s name] position… So that I think just in terms of like people covering that sort of [patient] panel… It’s not that they’re [LST templates] not getting done, it’s just, you know, part of a bigger workload on everybody.

Another HBPC provider shared she was in “*survival mode*” and had limited time to engage in continuing education related to LST template completion due to large increases in patient census and an inability to hire additional staff in a timely manner. She shared:

I think we’re up to like almost 400 patients. And I mean, we were like 200 [previously]… they’ve hired another physician, but then you know, it’s like [when is] she gonna start?

Another factor hindering sustainability when not present included not having the time to integrate goals of care conversations and LST template completion into visits. Sites underscored the challenges of fitting LST template completion into HBPC admission visits, particularly lacking time to have high-quality goals of care conversations during the first visit. When asked about any ongoing challenges to completing LST templates, an HBPC clinician shared.

*From my perspective*,* it continues to be the amount of time it takes to do it*,* it does take a little bit of extra time. You know*,* with each new admission you’re doing a lot*,* on the new admissions anyway so it’s the time*.

In contrast, program expansion proved to be a neutral factor that neither hindered nor enabled sustainment of LST template completion practices at half of the programs experiencing growth. At these programs where expansion had a limited impact on sustainability, enabling factors such as integrating LST template completion into admissions and visit processes and the team continuing to view documentation of LST preferences as a high priority ameliorated the potential challenges to sustainment of practices that may come with both program expansion and staff turnover.

We are just madly admitting people or and have been for the past six months or so. So that’s a huge victory to me. I think that people are not letting it go and have been really proactive with new patients.

### Mixed impact or limited impact factors

Across sites, the degree to which non-champion staff members were engaged and supportive of PERSIVED varied, with this factor acting as enabling when present or having a hindering or neutral impact when minimally present or absent. Champions from sites with higher degrees of staff engagement underscored how involving peers and obtaining buy-in were integral to sustaining high rates of LST template completion. As one provider champion shared:

*The success of the PERSIVED project in regards to [site name]*,* it’s been regarding or related to everyone participating. You know*,* when we started this*,* we were in the 40s and now we’re high scoring in the in the 90th percentile*.

In contrast, a few sites felt that the lack of support for PERSIVED and non-champion engagement hindered sustainment. Most of these sites created a provider-driven process for ensuring documentation of LST preferences due to the challenges of getting all additional staff involved. One provider champion noted this challenge as follows:

*I still have failed at inspiring my nurses to be part of this at all…they feel that it’s the PCP’s role and they feel that they have enough other full reminders and things that they have to cover in their own visit.*

Two factors that universally had neutral impacts on sustainability across sites were continuing education and use of PERSIVED resources. When asked if any continuing education on topics related to LST template completion occurred during sustainability, champions consistently shared that no education had occurred, in part because their rates had remained stable or because LST template completion had become a standard of care. As one champion shared:

I didn’t feel like there was a need to, like, have any, you know, like reminders or new, like, refresher sessions because our numbers have continued to be so good.

Similarly, champions reported minimal to no usage of the PERSIVED intervention resources such as training, sustainability plans, and process maps during sustainability, and this did not appear to have an impact on sustainability.

## Discussion

In this evaluation, we explored factors influencing sustainment of the PERSIVED evidence-based practice across VA HBPC programs. Our quantitative data indicated that all sites maintained or exceeded their LST documentation rates achieved during implementation. Using the MMCs approach and the CSAT as a guiding framework, we identified factors that help explain barriers and facilitators to sustainability among sites that participated in PERSIVED.

A number of factors relating to integration of PERSIVED into existing workflows and practices emerged as enabling factors that facilitated sustainment of the evidence-based practice. These factors included embedding LST documentation into existing processes, integrating review of LST documentation into regular meetings, and the behavior becoming an automatic practice that HBPC team members were expected to perform as a standard part of patient care. Prior work within health care settings suggests that incorporating evidence-based practices into standard practice and existing routines facilitated sustainment, and inadequate integration emerged as barriers to sustainability [12, 17–19]. Relatedly, PERSIVED sites that included LST template completion on HBPC provider performance reviews identified this as contributing to maintenance of high LST documentation rates and enforcing expectations to complete LST templates. Another study that explored determinants of program sustainability within the VA reported that the lack of performance expectations for evidence-based practices hindered sustainability [20]. While many PERSIVED sites described how integrating LST template completion into routine practices helped with sustainability, limited time to elicit and document Veteran preferences during visits and the time required to complete the template emerged as hindering factors when present. These findings echo previous studies in which time constraints emerged as a key determinant to sustainability [11, 12].

PERSIVED champions underscored the positive influence of leadership support and engagement on their ability to sustain LST template completion rates. Myriad studies have identified leadership support as a critical factor to sustaining evidence-based practices [8, 10–12, 17]. Notably, a systematic review of factors contributing to the sustainability of health care programs found that half of studies identified leadership support as a determinant of sustainability [21]. At many PERSIVED sites, ongoing leadership support contributed to LST preference documentation remaining a high priority during the sustainability period. Prior work by our team has shown that champions who believe in the evidence-based practice, are committed to the program, are motivated to serve in the champion role, and are engaged with their peers demonstrate successful adoption during the implementation phase [22]. Beyond leadership engagement, site champions noted varying levels of staff buy-in and engagement during sustainability. While having HBPC team members who believed in the importance of completing LST templates was an enabling factor when present and a hindering factor when absent, the degree to which non-champion staff were engaged in goals of care conversations and documenting LST preferences was not a clear determinant of sustainability. A paper by Sullivan and colleagues about an intervention conducted in VA nursing homes similarly noted varying levels of site staff buy-in and engagement that did not perfectly correlate with high and low sustainment of a dementia behavior evidence-based practice [19]. In contrast, other implementation studies conducted in the VA clearly identified the lack of staff buy-in, awareness, and interest as substantial barriers to program sustainment [12, 20].

Providing training and continuing education to existing staff emerged as a neutral factor neither enabling nor hindering PERSIVED efforts. Similarly, champions reported minimal use of PERSIVED intervention resources and sustainability plans and indicated lack of use of these resources had no impact on sustainability. In contrast, prior work on sustainability emphasizes the facilitating impact of ongoing education and sustainment planning on ongoing implementation of evidence-based practices [10, 12, 17, 23]. Across PERSIVED sites, baseline knowledge of advance care planning was relatively high, and coaching did not focus on advance care planning education. Rather, PERISVED coaching aimed to improve awareness of an appropriate process to document LST preferences, to help champions develop processes to complete goals of care conversations and LST templates, and to support integration of these processes into standard practices. This focus on making process changes during implementation rather than addressing knowledge gaps may help explain why ongoing education and training had a neutral impact on sustainability.

While training of existing staff did not occur frequently during the PERSIVED sustainability period, participants at many sites viewed integrating training on LST preference documentation into orientation for new hires as an enabling factor to sustaining current LST completion practices. Despite plans to train new employees, several PERSIVED sites highlighted how inadequate staffing and an inability to hire new staff in a timely manner hindered their ability to have dedicated time to conduct goals of care conversations and complete LST templates. These barriers were exacerbated for HBPC programs with a program census expansion during sustainability and some sites identified program growth as another factor hindering sustainability. Within prior work, lacking personnel, staff turnover, and champion turnover have commonly been identified as barriers to sustainability of health care interventions [2, 12, 21, 23].

When asked about use of the PERSIVED feedback report data during sustainability, most champions indicated that they continued to review and share these data, and that this practice supported continuing to document LST preferences and identify Veterans who still needed LST templates. Due to the regular use of feedback report data, a couple of sites developed mechanisms to monitor LST template completion after PERSIVED ended. However, the majority of PERSVIED sites lacked a system or plan for monitoring their data and felt this would hinder long-term sustainability given they would not be able to track ongoing progress or address lapses in future performance. A systematic review of program stability in health care settings indicated that receiving feedback on program performance and outcomes was a facilitator to sustainability [21]. Moreover, an implementation study by Bravata and colleagues found that one of the factors that was most associated with sustainability of an evidence-based stroke intervention was creating a system for sites to obtain and review data on their ongoing performance [17]. Based on feedback from PERSIVED champions on the need for a mechanism to monitor LST template completion data, the PERSIVED team collaborated with National HBPC leadership to add the feedback report data to an existing online dashboard utilized by VA HBPC programs. As this dashboard is already integrated into standard HBPC program practices, there is a greater likelihood of use moving forward and the potential for these data to meet the needs identified by PERSIVED champions [24].

Taken together, our findings indicate several strategies to enable the sustainment of interventions implemented within health care settings. First, we suggest working with teams to integrate interventions into existing processes and identify key roles of those involved in making it a standard of care. Second, if an intervention involves reporting on performance data, it is beneficial to generate performance data utilizing existing infrastructure and a method that can be continued independently after the project ends. Third, we suggest encouraging sites to weave the intervention into team meetings and performance reviews. Finally, given the differentiating nature of team awareness and leadership engagement, we suggest intervention teams make efforts to involve leadership and individuals beyond site champions throughout the intervention.

There are several limitations to our analysis of sustainability across HBPC programs enrolled in PERSIVED. First, we conducted sustainability interviews with PERSIVED champions and leadership and did not capture perspectives from other HBPC team members not in champion roles. Additionally, we could not conduct interviews with champions from one PERSIVED program during the sustainability phase because the program ceased participation during the implementation phase. These champions had left their HBPC program prior to sustainability. We purposefully recruited sites to participate based on baseline LST template completion rates and feedback from the national HBPC program director. This approach introduced recruitment bias as sites that were invited to participate may be different than those that were not selected to participate. Moreover, there are limitations to the generalizability of our results as the participating HBPC programs may not be generalizable to other programs that did not participate or to non-VA home based primary care settings. Several findings could benefit the implementation of evidence-based practices in health care settings outside of VA. The importance of integrating the new requirement into routine workflows, providing performance data and reinforcing expectations by leadership are all transferrable to other settings. These findings may all be beneficial to settings that are trying to prioritize the documentation of preferences for life-sustaining treatments.

## Conclusion

Sustaining evidence-based practices in health care requires more than just successful initial implementation; it depends on whether new practices can become embedded in routine clinical work and supported by organizational structure. All VA HBPC programs participating in PERSIVED maintained or exceeded the LST documentation rates that were achieved during the implementation phase. Our findings suggest that sustainment was supported by integration of LST documentation into routine workflows, continued use of audit with feedback, and reinforcement of expectations through leadership engagement and team awareness of the importance of documenting life-sustaining treatment preferences.

Our work highlights sustainability of evidence-based practices as an area for further research. Although we identified factors associated with sustainability across sites, we could not determine which determinants were most influential. Future studies could examine sustainability over a longer period to better understand factors key to long-term sustainment of new standard practices, as prior work has suggested that longer-term sustainability begins two years after the implementation period [8]. Future work could also explore the distinction between sustainment and institutionalization, including whether practices continue to require active implementation support or have become fully embedded as standard of care. Future research could explore how data gathered during the pre-implementation and implementation phase informs implementation strategy development, and in turn, affects sustainment. Such work could refine strategies to ensure that improvements in care delivery are sustained over time.

## Supplementary Information

Below is the link to the electronic supplementary material.

Supplementary Material 1

Supplementary Material 2

## Data Availability

The dataset generated and analyzed during the current study are not publicly available due to confidentiality reasons but are available from the corresponding author upon reasonable request.

## Abbreviations

CSAT: Clinical Sustainability Assessment Tool
CDW: Corporate Data Warehouse
GoCC: Goals of care conversation
HBPC: Home Based Primary Care
IDT: Interdisciplinary Team
LST: Life-sustaining treatment
LSTDI: Life-Sustaining Treatment Decisions Initiative
MMC: Matrixed multiple case
NCEHC: National Center for Ethics in Health Care
OPPE: Ongoing Professional Practice Evaluation
PERSIVED: Preferences Elicited and Respected for Seriously Ill Veterans through Enhanced Decision-Making
VA: Department of Veterans Affairs
