# Supplementary material for: Beyond implementation: what drives long-term sustainment of life-sustaining treatment documentation in VA home based primary care
Source: BMC Health Serv Res. 2026 Jul 9;26:1355. doi: 10.1186/s12913-026-15043-7 (PMC13637382; doi:10.1186/s12913-026-15043-7)
Supplement: Supplementary file 1 [file 12913_2026_15043_MOESM1_ESM.docx]

**Additional File 1: PERSIVED Sustainability Interview Guides**

**PERSIVED QUERI Sustainability Focus Group Questions (4 Months)**

Hello [interview participant name],

My name is [interviewer name] and this is my colleague [name]. We are with the PERSIVED QUERI program and the purpose of the call today is to gather feedback about your participation in the sustainability phase of the program. As you’ll remember, the purpose of your participation in PERSIVED was to increase completion of the life-sustaining treatment decisions template amongst your Home Based Primary Care Veterans while also ensuring Veterans have high quality of Goals of Care Conversations. Your feedback today will help us to improve the program. The interview will take approximately 30 minutes.

Do you have any questions before we begin?

In order to make sure we capture all of the information you provide, we would like to record this interview. The audio-file for the recording will be uploaded to a restricted access file on the VA intranet. Is this okay with you?

1. Since our last call, how have things been going with completing LST templates? Looking at your most recent feedback report, you are at [enter percentage].
   a. What has worked well?
   b. What have you found challenging?
   c. Have there been any changes on your team that have impacted LST template completion rates?
2. Tell us about how you have used our feedback reports and rosters since we talked to you last.
3. During our last few coaching calls, we filled in a sustainability action plan together. Tell us how, if at all, you have used the plan?
   a. How will continue to monitor your data once feedback reports stop (i.e. VSSC reports, from roster they maintain)?
   b. How are you training new staff on LST template completion and GoCC?
   c. What, if any, plans do you have for continuing education for existing staff?
4. Tell us about any other changes you have made to sustain improvements in LST template completion since we last talked.
   a. Can you provide a specific example?
5. Compared to other initiatives, how high of a priority is LST template completion currently in your HBPC program?
   a. Tell us about leadership involvement in LST template completion.
6. Is there anything else you would like to add ?

Thank you so much for participating. Do you have any questions for us?

**PERSIVED QUERI Sustainability Focus Group Questions (12 Months)**

Hello [interview participant name],

My name is [interviewer name] and this is my colleague [name]. We are with the PERSIVED QUERI program and the purpose of the call today is to gather feedback about your participation in the sustainability phase of the program. As you’ll remember, the purpose of your participation in PERSIVED was to increase completion of the life-sustaining treatment decisions template amongst your Home Based Primary Care Veterans while also ensuring Veterans have high quality of Goals of Care Conversations. Your feedback today will help us to improve the program. The interview will take approximately 30 minutes.

Do you have any questions before we begin?

In order to make sure we capture all of the information you provide, we would like to record this interview. The audio-file for the recording will be uploaded to a restricted access file on the VA intranet. Is this okay with you?

7. Since our last call, how have things been going with completing LST templates? Looking at your most recent feedback report, you are at ___ %.
   a. What has worked well?
   b. What have you found challenging?
   c. Have there been any changes on your team that have impacted LST template completion rates?
8. Since the coaching calls ended, have continued to engage in activities to promote LST template completion for your Veterans?
   a. Have you met as champions or with team members or leadership to discuss LST template completion?
   b. If yes, how often, is it helpful, has anyone else from the team been involved?
   c. If no, why not?
9. Tell us about how you have used our feedback reports and rosters since we talked to you last.
   a. The feedback reports and rosters will no longer be sent now that the PERSIVED program is ending. Do you have plans for how you will continue to monitor this data? (i.e. VSSC reports, from roster they maintain)?
10. During our last few coaching calls, we filled in a sustainability action plan together. Tell us how, if at all, you have used the plan?
    a. How are you training new staff on LST template completion and GoCC?
    b. What, if any, plans do you have for continuing education for existing staff?
11. Tell us about any other changes you have made to sustain improvements in LST template completion since we last talked.
    a. Can you provide a specific example?
    b. Are there any changes to processes that you created while working with PERSIVED that you’ve now stopped?
       i. If yes, why?
12. How confident do you feel about your team’s ability to sustain improvements in LST template completion?
    a. Why do you feel this way ?
13. Compared to other initiatives, how high of a priority is LST template completion currently in your HBPC program?
    a. Tell us about leadership involvement in LST template completion.
    b. Has LST template completion been added to OPPE for HBPC providers?
14. Is there anything else you would like to add?

Thank you so much for participating. Do you have any questions for us?
