# Supplementary material for: Beyond implementation: what drives long-term sustainment of life-sustaining treatment documentation in VA home based primary care
Source: BMC Health Serv Res. 2026 Jul 9;26:1355. doi: 10.1186/s12913-026-15043-7 (PMC13637382; doi:10.1186/s12913-026-15043-7)
Supplement: Supplementary file 2 [file 12913_2026_15043_MOESM2_ESM.docx]

**Additional File 2: PERSIVED Analytic Plan**

**Comparing Variations in LST template completion in implementation and sustainability across PERSIVED sites**

The approach is based on the paper by Kim et al which “outlines the matrixed multiple case study approach, which allows for understanding how these processes and influences similarly or differently interact with outcomes across multiple implementation sites.”

| Step | Steps from Kim et al. | Steps for PERSIVED |
| --- | --- | --- |
| 1 | Establish research goal | We aim to understand barriers and facilitators to sustainability at PERSIVED sites and the impact on these factors on site LST rates during the 12-months after implementation. |
| 2 | Define the dependent variable  (i.e., the dependent variable) | Successful adoption of practice change during implementation: Reaching 80% LST completion rate during the implementation period (achieved).  Sustainability of practice change 1-year after implementation: LST completion rate at the end of sustainability is within 5% of highest rate during implementation (stable sustainability) or greater than 5% improvement (high sustainability) |
| 3 | Select domains of factors influencing implementation  (independent variables) | We used the clinical sustainability assessment tool/framework to guide selection of relevant domains. We selected six of the seven CSAT domains:   1. Engaged Staff & Leadership; 2. Monitoring & Evaluation; 3. Implementation & Training; 4.Workflow Integration; 5. Outcomes & Effectiveness; 6.Organizational Readiness |
| 4 | Gather data on both implementation success (the dependent  variable) and potentially relevant domains (the independent variables) | We conducted sustainability group interviews at two times during the 12 months of sustainability.  Sustainability group interviews were analyzed for content based on a priori codes from the interview guide and deductive codes that emerged. Subsequently, we organized codes into code groups corresponding with the CSAT domains listed in step 3. Data for the dependent variable (LST rates) is from feedback reports. |
| 5 | Assess the extent of implementation success (the dependent  variable) per site | We assessed performance during implementation and sustainability per site to categorize the ten PERSIVED sites into four categories based on LST completion rates. These categories were defined as:  **Achieved Implementation & Steady Sustainability** (reached 80% LST completion during implementation & rate at end of sustainability is within 5% of highest rate achieved during implementation)  **Achieved Implementation & High Sustainability** (reached 80% LST completion during implementation & rate at end of sustainability is 6% or greater than the highest rate achieved during implementation)  **Did not achieve implementation & Steady Sustainability** (did not reach 80% LST completion during implementation & rate at end of sustainability is within 5% of highest rate achieved during implementation)  **Did not achieve Implementation & High Sustainability** (did not reach 80% LST completion during implementation & rate at end of sustainability is 6% or greater than the highest rate achieved during implementation) |
| 6 | Identify influencing factors (the independent variables) per data  source per site | We aimed to identify factors impacting sustainability at each site by generating code summaries for each site. Specifically, we limited the scope of the queries to a specific site and ran co-occur queries with the CSAT domain code groups and the facilitator and barrier code. We also created code summaries for distinct codes without using co-occur (i.e., good quote, feedback reports, incentives, sustainability plan, priority) that were not commonly double coded with the facilitator or barrier code. |
| 7 | Organize the examined data into a sortable matrix | We created an excel matrix with the CSAT domain code groups and the codes within each domain as rows and the PERSIVED sites as columns. |
| 8 | Conduct within-site analysis of matrixed data | Two analysts reviewed code summaries for each site. Analysts independently reviewed summaries for each site and indicated in the excel matrix the presence of each factor (somewhere on the scale from present to minimally present) and the impact of the factor on sustainability (enabling/facilitator, no impact, hindering/barrier) The analysts met to review their individual coding for each site and come to consensus. Minimally present \| \| \| \| \| \| Present \| N/A (not mentioned); Hindering \| ~~ \| ~ \| o \| * \| ** \| Enabling \| |
| 9 | Conduct cross-site analysis of data  “assessing whether factors and their combos  were (i) present across multiple sites, (ii) consistently  associated with higher/ lower sustainability,  and (iii) emphasized at some sites more than others.” | Once the matrix is assembled, analysts will explore which determinants under the CSAT domains are common across PERSIVED sites as both barriers and facilitators, which determinants are related to high sustainability vs. steady sustainability, and which factors are different between sites.  Universal enabling factors: look for which factors are present across multiple sites and are commonly enabling.  Differentiating factors: which factors or valence are linked to higher vs. lower outcomes across sites (e.g. enabling in high sites, hindering in low sites).  Sort by domain to identify trends and sort by presence and hindering/enabling. |
